# Less is more: reconsidering asymptomatic *Chlamydia trachomatis* and *Neisseria gonorrhoeae* screening for gay, bisexual and other men who have sex with men in the era of doxycycline post-exposure prophylaxis (DoxyPEP)

**DOI:** 10.1177/09564624261481866

**Published:** 2026-08-26

**Authors:** David James Field, Johnny Boylan, Fiona Lyons, Ralph Hurley O’Dwyer, Chantal Den Daas

**Affiliations:** 1 Health Psychology Group, 1019 University of Aberdeen, Aberdeen, UK; 2 Gay Men’s Health Service, 8004 Health Service Executive, Dublin, Ireland; 3 Department of Infectious Diseases and Genitourinary Medicine, St James’s Hospital, Dublin, Ireland; 4 Department of Public Health, HSE Dublin and Midlands, 8004 Health Service Executive, Dublin, Ireland

**Keywords:** gbMSM, chlamydia, gonorrhoea, asymptomatic screening, DoxyPEP, syphilis, antimicrobial stewardship, overdiagnosis, risk, PrEP

## Abstract

Routine high-frequency screening for asymptomatic *Chlamydia trachomatis* and *Neisseria gonorrhoeae* among cisgender gay, bisexual and other men who have sex with men (gbMSM) is established despite limited evidence that it reduces population-level incidence or harm. Recent studies support reconsideration but do not settle the balance of benefits and harms. Reducing screening could delay diagnosis and affect transmission or partner notification; continuing screening may increase antimicrobial exposure, possible overdiagnosis, anxiety and stigma. The demonstrated benefits and unresolved long-term stewardship implications of doxycycline post-exposure prophylaxis (DoxyPEP) illustrate why antimicrobial use should be differentiated. We argue for cautious, co-produced, evaluated and reversible de-implementation while maintaining symptomatic, contact-based and otherwise clinically indicated testing, HIV and syphilis screening and HIV pre-exposure prophylaxis (PrEP) care. With transparent communication and community partnership, less screening need not mean less care.

## Introduction

Routine high-frequency screening for asymptomatic *Chlamydia trachomatis* (CT) and *Neisseria gonorrhoeae* (NG) among cisgender gay, bisexual and other men who have sex with men (gbMSM), particularly those using HIV pre-exposure prophylaxis (PrEP), became embedded in guidelines and clinical practice on the plausible assumption that detecting and treating infection would reduce incidence and harm. However, this practice was normalised before direct trial evidence established population-level benefit. Contemporary screening principles require adequate understanding of a condition’s natural history and evidence that the overall benefits of a screening programme outweigh its harms.^1^

This distinction matters because diagnostic testing responds to symptoms, contacts or clinical concerns, whereas screening actively invites people without symptoms to be tested in the expectation that earlier detection will improve outcomes. The number of infections detected is not, by itself, evidence that screening is beneficial. Detection yield, individual clinical benefit and population-level impact are related but distinct outcomes.

Gilmore et al. argue that decisions to reduce screening should not be guided by epidemiology and antimicrobial stewardship alone because testing can also provide reassurance, sustain healthcare engagement and support trust.^2^ We agree that testing is socially meaningful; however, its possible harms must be included in the same analysis. The debate is not confined to gbMSM: Dutch policy has reduced asymptomatic chlamydia testing, while the 2025 European guideline discusses the limited value of broad screening in asymptomatic populations.^3,4^

Here, “low-value screening” means screening of uncertain individual or population benefit, where harms or resource use may outweigh benefits. We address routine asymptomatic CT/NG screening, not symptomatic, contact-based or clinically indicated testing; individualised testing following informed discussion; partner management; HIV or syphilis testing; PrEP care; or DoxyPEP. We question default three-site, three-monthly screening, not all asymptomatic testing.

## Evidence, uncertainty and potential harms

A systematic review of observational studies found little evidence that asymptomatic CT/NG screening among gbMSM reduced prevalence.^5^ Evidence remains limited and does not establish that reducing screening is harmless. WHO now conditionally recommends screening asymptomatic MSM, while rating the certainty of evidence as low and noting that the optimal screening frequency remains unclear.^6^

The GonoScreen randomised trial is similarly nuanced. Combined CT/NG incidence was 0.155 cases per 100 person-days with three-site, three-monthly screening and 0.205 without screening. Non-screening did not meet the prespecified non-inferiority criterion and was associated with higher CT incidence, including symptomatic CT. However, screening did not reduce NG incidence and was associated with greater antimicrobial consumption.^7^ A subsequent Belgian cohort following reduced screening reported lower antimicrobial consumption without an increase in symptomatic infections.^8^ Collectively, these findings support re-evaluation, not the conclusion that the issue is settled.

Reduced screening could delay diagnosis, alter transmission within highly connected sexual networks, affect partner notification or facilitate transmission to people at greater risk of complications. Effects may also differ by infection and anatomical site. De-implementation should therefore be cautious, evaluated and reversible, with explicit monitoring of these outcomes.

Possible overdiagnosis is one potential harm of continued intensive screening. We use the term narrowly to describe detection of an infection that would not have produced meaningful clinical or public-health harm, or whose detection and treatment do not improve outcomes. We acknowledge that applying overdiagnosis to infectious diseases is contested because untreated infections may be transmitted.^9^ Nevertheless, nucleic acid amplification tests detect pathogen nucleic acid without establishing likely duration, transmissibility or clinical consequence. These questions are particularly relevant to asymptomatic extragenital infections in men, for which long-term sequelae are less clearly established than those associated with cervical infection.

Spontaneous clearance further complicates the assumption that every asymptomatic diagnosis has equivalent significance. Mensforth et al. observed spontaneous clearance in 20.5% of gonorrhoea infections over a median interval of 10 days, while the NABOGO analysis reported clearance in 14.5% of anal, 18.7% of pharyngeal and 32.1% of urethral infections.^10,11^ These findings neither make detected infections unreal nor justify withholding indicated care; they demonstrate uncertainty about the benefit produced by detecting and treating every asymptomatic infection.

## Surveillance and the production of risk

Routine screening does not simply reveal a pre-existing burden of infection; it also helps produce the epidemiological picture through which gbMSM are classified as high risk. All else being equal, populations tested more frequently and at more anatomical sites will have more infections detected. This is not a flaw in surveillance, but a condition of how surveillance data are produced. Modelling illustrates the resulting “testing paradox”: observed STI prevalence may increase while true prevalence falls as testing intensity rises.^12^ A circular process may therefore emerge: risk classification justifies intensive screening; intensive screening generates more diagnoses; and those diagnoses reinforce the original classification.

These classifications may have material and psychosocial consequences. They may support access to services and prevention resources, but they may also position gbMSM as inherently risky or requiring exceptional biomedical monitoring, thereby shaping clinical expectations, public-health messaging and community self-understanding.

This is a conceptual and ethical interpretation rather than a claim that these psychosocial effects have been empirically established. A conceptual review found that risk in sexual-health research involving gbMSM is heterogeneous and frequently imprecisely defined.^13^ Routine screening may translate these situated and uncertain experiences into apparently objective clinical categories. An interpretative phenomenological study further found that Irish gay men understand sexual-health risk through embodied, emotional and cultural meanings rather than biomedical probability alone.^14^ Screening may therefore provide reassurance while also shaping how risk is understood and reproducing the expectation that asymptomatic gay sex requires continuous biomedical verification.

## DoxyPEP and differentiated stewardship

DoxyPEP illustrates why antimicrobial stewardship should be differentiated rather than invoked as a blanket objection to antibiotic-based prevention. Randomised trials among gbMSM and transgender women at increased risk of bacterial STIs have demonstrated substantial reductions in syphilis and chlamydia, with more variable protection against gonorrhoea.^15–17^ These are clinically meaningful prevention benefits, particularly for syphilis.

However, DoxyPEP is not without uncertainty. Long-term selection for antimicrobial resistance in NG, commensal organisms and other potential pathogens, together with wider ecological effects, remains under investigation.^18^ A six-month substudy found no significant change in overall gut microbiome diversity or composition, but identified increases in the proportion and expression of tetracycline-resistance genes.^19^ The clinical significance and longer-term implications of these findings remain uncertain. DoxyPEP should therefore be targeted to those most likely to benefit, reviewed regularly and accompanied by resistance surveillance.

The appropriate stewardship question is not whether antibiotic exposure is always harmful, but where its expected benefits justify its risks. Antibiotic use with demonstrated prevention benefits should not be conflated with exposure arising from screening whose net benefit remains uncertain.

This requires separating HIV, syphilis, CT and NG rather than treating them as interchangeable targets of “routine STI screening”. They differ in natural history, clinical consequences, transmission and available prevention tools. A coherent strategy may therefore increase investment in syphilis prevention and appropriately targeted DoxyPEP while reducing CT/NG screening that does not demonstrate comparable benefit.

## Community-conscious de-implementation

Reassurance, continuity and trust matter. Qualitative studies suggest that some gbMSM may interpret reduced screening as a cost-cutting measure or evidence of declining concern for their health and may initially be hesitant about change. Greater acceptance is possible when the rationale, benefits and possible harms are communicated transparently by trusted healthcare professionals.^20,21^ However, communication alone is insufficient. Reduced screening should be co-produced by communities, clinicians, service providers and policymakers jointly evaluating the rationale for change, agreeing the scope and outcomes of any pilot and the circumstances in which the approach should be modified or reversed. Co-production does not require indefinite continuation of a familiar practice without adequate evidence of benefit; it requires that decisions about how practices are evaluated and changed are made transparently with, rather than simply communicated to, affected communities.

As a conceptual and ethical consideration, testing can operate as a norm of responsible sexual citizenship: regular screening becomes evidence of responsibility, while testing less may be interpreted as carelessness even when clinically appropriate. For gbMSM, this may reproduce broader expectations of disclosure, testing, prevention and treatment adherence. Community engagement should create space to examine these norms, not merely preserve them.

A community-conscious approach would maintain accessible symptomatic, contact-based and clinically indicated testing; access to individualised testing following informed discussion; partner management; HIV and syphilis testing; PrEP monitoring; indicated DoxyPEP; and broader sexual-health support. It would monitor symptomatic disease, transmission, antimicrobial consumption and resistance alongside service engagement, understanding, trust, stigma and inequalities. Any evidence of material harm should prompt review.

Careful language is essential. Reduced screening should never be framed as “gbMSM test too much” or “gay men are driving antimicrobial resistance”. Individuals followed testing practices created and encouraged by health systems. Responsibility for revising those practices transparently therefore rests with health systems, not communities.

## Conclusion

The choice is not between epidemiology and community, or between screening and abandonment. It is whether frequent routine asymptomatic *C.trachomatis / N.gonorrhoeae* screening provides sufficient benefit to justify its possible clinical, antimicrobial and psychosocial harms. The current evidence supports cautious re-evaluation while recognising uncertainty in both directions. With maintained access to comprehensive sexual healthcare, co-produced implementation, transparent communication and robust monitoring, less screening need not mean less care.
